# Development of Green and Efficient Extraction of Bioactive Ginsenosides from *Panax ginseng* with Deep Eutectic Solvents

**DOI:** 10.3390/molecules27144339

**Published:** 2022-07-06

**Authors:** Yujia Tu, Linnan Li, Wenxiang Fan, Longchan Liu, Zhengtao Wang, Li Yang

**Affiliations:** 1 The MOE Key Laboratory of Standardization of Chinese Medicines, The SATCM Key Laboratory of New Resources and Quality Evaluation of Chinese Medicines, Shanghai Key Laboratory of Compound Chinese Medicine, Institute of Chinese Materia Medica, Shanghai University of Traditional Chinese Medicine, Shanghai 201203, China; tuyujia_1997112@126.com (Y.T.); fwx13990706098@163.com (W.F.); liulongchan@shutcm.edu.cn (L.L.); ztwang@shutcm.edu.cn (Z.W.); 2 Shanghai Frontiers Science Center of TCM Chemical Biology, Institute of Interdisciplinary Integrative Medicine Research, Shanghai University of Traditional Chinese Medicines, Shanghai 201203, China

**Keywords:** deep eutectic solvents, ginsenosides, *Panax ginseng*, ultrasound-assisted extraction

## Abstract

The extraction of active constituents from natural sources in a green and efficient manner is considered an important field in the pharmaceutical industry. In recent years, deep eutectic solvents (DESs), a new type of green solvent, have attracted increasing attention. Therefore, we aimed to establish a green and high-efficiency extraction method for ginsenosides based on DESs. This study takes *Panax ginseng* as a model sample. Eighteen different DESs were produced to extract polar ginsenosides. Ultrasound-assisted extraction (UAE) was applied for simplicity and efficiency. A binary DES synthesized using choline chloride and urea at a proportion of 1:2 prepared by a heating stirring method is proven to be more effective than other solvents, such as the widely used 70% ethanol for the extraction of ginsenosides. Three variables that might affect the extraction, including the DES content in the extraction solvent, liquid/solid ratio, and ultrasound extraction time, were evaluated for optimization. The optimum extraction conditions for ginsenosides were determined as follows: DES water content of 20 wt%, liquid/solid ratio of 15 mL g^−1^, and an ultrasonic extraction time of 15 min. The extraction yield for the optimized method is found to be 31% higher than that for 70% ethanol, which achieves efficient extraction. This study shows that DESs are available to extract ginsenosides for use in traditional Chinese medicine. The discovery also contributes to further research into the green extraction of ginsenosides.

## 1. Introduction

In recent years, the contribution of natural active components such as ginsenosides, triterpenes, flavonoids, and phenolic acids to human health has attracted increasing interest. In addition to their reported pharmacological effects, including antioxidative, anti-inflammatory, and anticancer properties, biologically active compounds play an important role in pharmaceutical quality control [1,2,3,4]. Thus, the extraction of active ingredients is critical. At present, organic solvents are still commonly applied to extract active components from plant materials [5]. However, the toxicity and volatility of organic solvents pose a threat to human health and the ecological environment [6,7]. Therefore, the development of green solvents that can replace conventional organic solvents is imperative. Ionic liquids (ILs) are organic salts that are liquid at room temperature and consist of organic cations and inorganic or organic anions. Their melting points are below the boiling temperature of water (100 °C). Due to their negligible volatility and low flammability, ILs are qualified to be an alternative to organic solvents. However, several reports have pointed out the shortcomings of ILs, which are detrimental to their “greenness”, including poor biodegradability, high cost of synthesis, and harmful toxicity [8,9,10,11,12]. Therefore, the development of a solvent that can replace traditional organic solvents and ILs while having similar properties to ILs is of great significance.

Deep eutectic solvents (DESs) were proposed by Abbot et al. in 2003 as substitutes for organic solvents and ILs [13]. DESs are a type of eutectic mixture formed by hydrogen bond acceptors (HBA) and hydrogen bond donors (HBD) through strong hydrogen bond interactions. Regarding the composition of DESs, HBAs are usually quaternary ammonium salts, and the most typical is choline chloride (ChCl). HBDs are usually amides, carboxylic acids, alcohols, and amino acids [14,15,16]. Compared to HBAs and HBDs on their own, a DES has a much lower melting point. Under most circumstances, DESs are liquids between ambient temperature and 70 °C. As eutectic mixtures, the eutectic points of DES mixtures are lower than those of ideal liquid mixtures. Due to their biodegradability, low synthetic cost, and nontoxicity, DESs are superior to ILs. Furthermore, DESs have adjustability, negligible fluctuations, and an extensive polarity range [15,16]. DESs have been employed in all kinds of study domains, including extraction processes and organic syntheses. Previous studies have investigated DESs and established various methods for extracting bioactive components from natural products [17,18,19,20,21,22,23,24,25].

Generally, the commonly used methods for extracting active ingredients include heating reflux extraction (HRE), microwave-assisted extraction (MAE), supercritical fluid extraction (SFE) and ultrasound-assisted extraction (UAE) [21,22,23,26], which is one of the most applied extraction technologies. Ultrasound is a key technology to achieve sustainable “green” extraction targets. With this technique, extraction can be accomplished in a short time with high reproducibility, while simplifying operations and posttreatment, reducing solvent consumption, improving the purity of the final product, and expending only a small amount of fossil energy compared to that required by conventional extraction methods [27,28]. In this study, we chose UAE as the extraction method to investigate DES-based extraction of natural products.

To perform the investigation, *Panax ginseng* was chosen as a model plant. As an important herbal plant, *P. ginseng* root has been used for the health care and treatment of various diseases, such as coma, gastrointestinal disease, and cardiovascular disease. Ginsenosides are considered to be the main active compounds of *P. ginseng* and have various pharmacological effects, such as anti-aging, anti-tumor, and anti-diabetes activities [29,30,31]. In addition, ginsenosides are usually used for the quality control of *Panax ginseng*. Therefore, ginsenosides were chosen as the target active ingredients to study green extraction methods based on DES-UAE. White ginseng was selected for this study because it has been reported to contain mostly major polar ginsenosides, such as Rg_1_, Re, Rf, Rb_1_, Rb_2_, Rc, and Rd [30,31]. The chemical structures of four types of ginsenosides are shown in Supplementary Materials Figure S1. There are many reported methods for extracting ginsenosides from white ginseng [30,31,32,33,34,35,36]. Generally, the extraction solvents applied in existing methods, including HRE, UAE, and MAE, are primarily organic solvents, in which methanol, ethanol, and their aqueous solutions are most commonly utilized. For instance, in 2015, Wei Wu et al. analyzed and identified 52 ginsenosides by UAE of 80% aqueous methanol (*v*/*v*) combined with a UHPLC–Q-TOF-MS method [34], while in 2017, Xin Huang et al. identified 59 ginsenosides via UAE of the same solvents combined with an HPLC–MS^n^-equipped electrospray ionization ion source [35]. In the previous research of our group, 70% aqueous methanol (*v*/*v*) was most commonly used [36]. Nevertheless, despite the high extraction efficiency of these methods, disadvantages, including the extensive use of organic solvents and long extraction times, still exist. Therefore, the development of green extraction methods for ginsenosides is of utmost importance. Throughout the current research reports on the extraction of ginsenosides by DES, there have only been 10 studies in the past five years, and most of them were about the transformation of ginsenosides. At present, only Jeong K M et al. in 2015 established a new DES-UAE method to extract ginsenosides from white ginseng. In their study, the freeze-drying method was used to prepare DES solvent, and ultrasonic-assisted extraction and high-performance liquid chromatography were applied to extract and analyze ginsenosides (ginsenosides Rg_1_, Re, Rf, Rb_1_ and Rc). In total, 24 kinds of DES solvents were designed and screened, and finally, a newly designed ternary DES solvent consisting of glycerol, L-proline, and sucrose (9:4:1) was proven to be the optimal solvent. The extraction effect of the new method was significantly higher than the reported solvents and extraction methods. This is the current optimal method for extracting ginsenosides from ginseng medicinal materials based on DESs [17]. However, the preparation of DESs required at least 24 h, and extraction and analysis took nearly 80 min. Therefore, the entire extraction procedure was complicated and time-consuming, which reduced the extraction efficiency.

The purpose of this research was to maximize the extraction efficiency of ginsenosides while simplifying the extraction procedure by DES-UAE. In this study, 18 different DESs were synthesized to extract ginsenosides from white ginseng. UAE was applied for simplicity and efficiency. UPLC-Q-TOF-MS and HPLC analytical systems were used for the qualitative and quantitative characterization of the extracted ginsenosides, respectively.

## 2. Experimental

### 2.1. Chemicals, Reagents, and Equipment

Choline chloride, sucrose, D-(+)-maltose monohydrate, D-(+)-glucose, glycerol, lactic acid, and L-proline were purchased from Sinopharm Chemical Reagent Co., Ltd. (Shanghai, China). Betaine, ethylene glycol, 1,2-propanediol, 1,4-butanediol, D-sorbitol, and urea were obtained from Adamas-beta (Shanghai, China). All the components of the DESs were of analytical grade. Methanol, acetonitrile, and formic acid of HPLC grade were obtained from Fisher Scientific (Santa Clara, CA, USA). HPLC-grade phosphoric acid was purchased from Honeywell (Seelze, Germany). Analytical-grade methanol and ethanol were purchased from Sinopharm Chemical Reagent (Shanghai, China). Ultrapure water was acquired via a Milli-Q water purification system (Millipore, Bedford, MA, USA). White ginseng powder was prepared from the roots of certified 3-year-old *Panax ginseng* by Prof. Zhengtao Wang (Institute of Chinese Materia Medica, Shanghai University of Traditional Chinese Medicine). Five reference compounds of ginsenosides Rg_1_, Re, Rf, Rb_1_, and Rc were purchased from Biopurfy Phytochemicals (Chengdu, China).

An electronic balance, model BT25S, was obtained from Sartorius Scientific Inc. (Beijing, China). A digital hot plate stirrer, model HMS-14, was obtained from Titan Scientific Co., Ltd. (Shanghai, China). A vortex mixer (model VX-200) was acquired from Labnet International Inc. (Edison, NJ, USA). An ultrasonic bath (model CPX5800H) was obtained from Branson (Danbury, CT, USA). A centrifuge (model 5424R) was obtained from Eppendorf (Hamburg, Germany). A Waters (Milford, MA, USA) OASIS HLB column (5 cc, 200 mg) was used as the SPE cartridge.

### 2.2. Preparation of Mixed Standard Solutions

Reference solutions of ginsenoside Rg_1_, Re, Rf, and Rb_1_ (2.0 mg mL^−1^) were produced in methanol and kept at 4 °C. A mixed standard solution of 0.4 mg mL^−1^ ginsenoside Rg_1_, Re, and Rb_1_ and 0.2 mg mL^−1^ ginsenoside Rf was prepared. Standard working solutions were prepared by diluting with methanol, with Rg_1_, Re, and Rb_1_ ginsenoside concentrations ranging from 25 to 400 μg mL^−1^, while the Rf ginsenoside concentration ranged from 12.5 to 200 μg mL^−1^.

### 2.3. Comparison of Different Conventional Solvents

Five conventional solvents, namely water, methanol, 70% methanol, ethanol, and 70% ethanol, were selected for comparison. For the extraction process, 100 mg of white ginseng powder was weighed and placed into a 1.5 mL microfuge tube. Afterwards, the prepared powder was soaked in 1.0 mL of solvent and briefly vortexed. The blend was sonicated for 45 min at ambient temperature and then centrifuged at 18,407× *g* for 15 min. The supernatant was separated and filtered through a 0.22 μm filter (ANPEL Laboratory Technology Inc., Shanghai, China) before HPLC-DAD analysis.

### 2.4. Preparation of DESs

In this study, DESs were produced via the heating and stirring method according to the literature [15]. Briefly, each component was weighed out and then mixed in a 150 mL beaker. After adding 10 mL of water, the mixture was magnetically stirred at 100 r min^−1^ and heated at 50 °C until a clear and transparent liquid was visible. All prepared DESs were sealed and chilled to ambient temperature and then stored at 4 °C. All generated DESs are enumerated in Table 1.

### 2.5. Green Extraction of Ginsenosides from Panax Ginseng Based on DESs

All the prepared DESs were blended with water at a ratio of 7:3 and applied to the primary screening of DESs. For the extraction process, 100 mg white ginseng powder was weighed and placed into a 1.5 mL microfuge tube. Afterwards, the prepared powder was soaked in 1.0 mL of solvent and briefly vortexed. The blend was sonicated for 45 min at ambient temperature and then centrifuged at 18,407× *g* for 15 min. The supernatant was separated and diluted fivefold with water and then applied to a HLB 5 cc cartridge that had been activated with equal volumes of methanol and water. After loading the DES extract, the column was rinsed with 5 mL water and 5 mL ethanol. The collected ethanol eluate was then evaporated at 60 °C in a water bath. Finally, the residues were redissolved with methanol and made up to 5 mL and then passed through a 0.22 μm filter (ANPEL Laboratory Technology Inc., Shanghai, China) before further qualitative and quantitative analysis.

### 2.6. Qualitative Analysis of Extracted Ginsenosides by UPLC-Q-TOF-MS Analysis

UPLC was performed by using a Waters ACQUITY UPLCTM system (Waters Corporation, Milford, MA, USA), which consisted of a binary solvent delivery system and an autosampler. A Waters ACQUITY HSS T3 column (100 mm × 2.1 mm, 1.8 μm) was utilized to separate the ginseng extracts. The column temperature was 45 °C, and the flow rate was 0.4 mL min^−1^. The injection volume was 2 µL. The mobile phase was composed of 0.1% formic acid in water (eluent A1) and acetonitrile (eluent B1). The gradient program was as follows: 0–2 min, 15–30% B1; 2–8 min, 30–35% B1; 8–10 min, 35–42% B1; 10–15 min, 42–44% B1; 15–21 min, 44–55% B1; 21–27 min, 55–95% B1; 27–29 min, 95% B1; and 29–30 min, 95–15% B1.

Mass spectrometry was performed by using a Waters G2 QTOF mass spectrometer, which was equipped with a negative mode electrospray ionization source. The full scan data were obtained from 100 Da to 1500 Da under the following conditions: cone voltage of 40 V, capillary voltage of −2.4 kV, desolvation temperature of 400 °C, source temperature of 120 °C, cone gas rate of 50 L/h, and desolvation gas rate of 800 L/h. The collision voltage was set at 25–50 eV. During data acquisition, the data were revised by utilizing an external reference, leucine enkephalin (*m*/*z* 554.2615). For the system control and data acquisition, MassLynx^TM^ software (Version 4.1, Waters, Milford, MA, USA) was utilized. On the basis of accurate mass measurements compared to the values reported in the literature, chromatographic peak identification and characterization of the ginseng extracts were performed.

### 2.7. Quantification of Extracted Ginsenosides by HPLC–DAD

HPLC-DAD analysis was conducted using an Agilent 1100, which was connected to a diode array detector (DAD) (Agilent Technology, Santa Clara, CA, USA). For the separation of compounds, an Accucore C_18_ column (150 mm × 4.6 mm, 2.6 μm) obtained from Thermo Fisher Scientific Inc. (Shanghai, China) was utilized. The column temperature was set at 25 °C, and the flow rate was 0.8 mL min^−1^. Meanwhile, the injection volume was 5 μL, and the detection wavelength was 203 nm. The mobile phase was composed of acetonitrile (eluent C) and water containing 0.1% phosphoric acid (eluent D). The gradient program was as follows: 0–7 min, 20% C; 7–20 min, 20–45% C; 20–35 min, 45–95% C; 35–40 min, 95% C. The system was equilibrated for 15 min before subsequent injections.

### 2.8. Investigation and Optimization of Extraction Conditions

The variables that may impact the extraction efficiency, namely the content of DES in the extraction solvent (20~80%), the liquid/solid ratios (5~25 mL g^−1^), and the ultrasonic extraction time (15~20 min), were evaluated and optimized. The extraction methods have been described in the relevant sections. The DES exhibiting the best performance in the quantification of ginsenosides was selected. Other conditions were the same as the original.

### 2.9. Method Validation

The linearity, sensitivity, precision, and extracted recovery of the established method were validated. The linearity was determined by mixing the standard solutions prepared above. The limit of detection (LOD) was determined according to the signal-to-noise ratio (S/N = 3), and the limit of quantitation (LOQ) was determined by the signal-to-noise ratio (S/N = 10). The precision test was performed using the same sample prepared from the established method within one day. The recovery was carried out by adding mixed standard solution to the accurately weighed white ginseng powder before extraction. The recovery was calculated using the following formula: Recovery (%) = (C − A)/B × 100%, where A represents the initial content of ginsenosides, B represents the added content of ginsenosides, and C represents the content determined by the HPLC-DAD method.

## 3. Results and Discussion

### 3.1. The Selection of Conventional Solvent and Active Compounds

The most commonly used ginsenoside extraction methods are organic solvents combined with UAE. Therefore, the five traditional solvents mentioned above were selected for the extraction of ginsenosides as reference solvents according to the literature [34,35,36]. According to the Chinese Pharmacopoeia [37], the index components for *Panax ginseng* are ginsenosides Rg_1_, Re, and Rb_1_. Based on quality standards, combined with our previous research [36], we chose five polar ginsenosides, Rg_1_, Re, Rf, Rb_1_, and Rc, to perform preliminary analysis. The HPLC chromatograms, peak areas, and extraction efficiencies for the five reference solvents are illustrated in Supplementary Materials Figure S2, Supplementary Materials Table S1, and Supplementary Materials Figure S3. The results demonstrate that 70% methanol and 70% ethanol show higher extraction efficiency. For environmental considerations, 70% ethanol was chosen for subsequent experiments. Water is more polar than methanol, and methanol is more polar than ethanol. Combined with the experimental results, it can be concluded that the polarity of the extraction solvent used has a remarkable effect on the solubility of ginsenosides.

**Figure 1:**
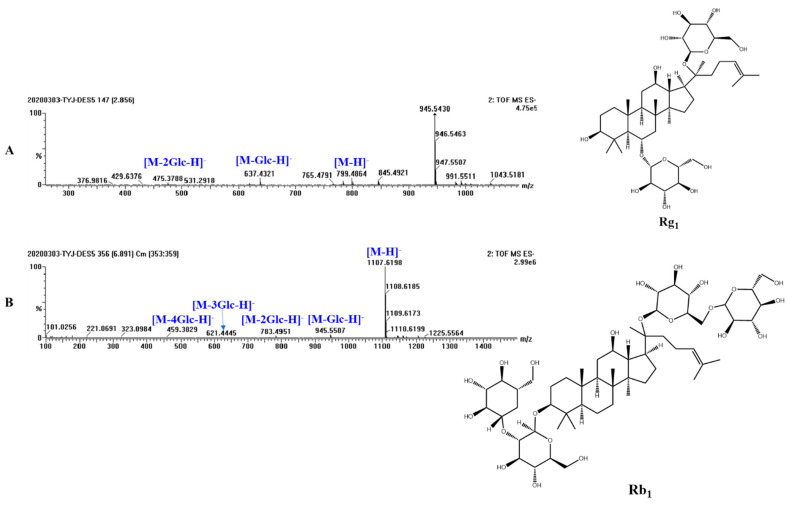
Secondary mass spectrum of representative types of ginsenosides of the DES5 extract ((**A**,**B**) are ginsenoside Rg_1_, ginsenoside Rb_1_).

**Figure 2:**
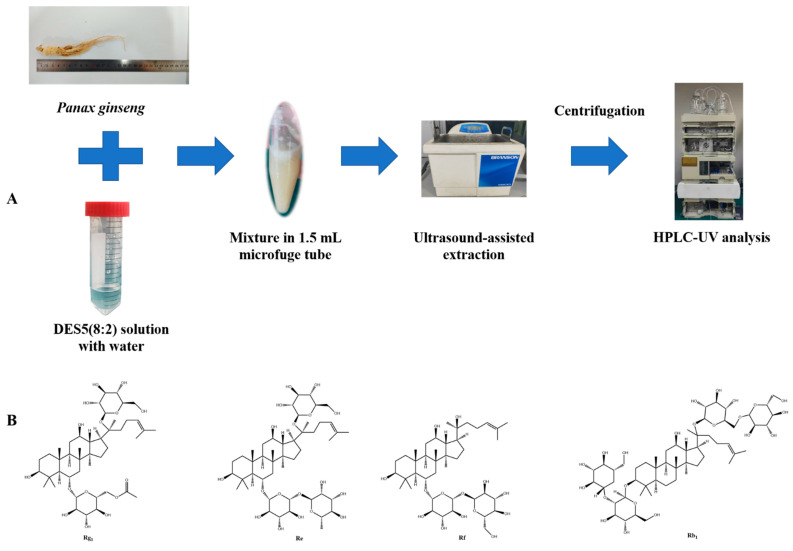
Overall scheme for deep eutectic solvent-based ultrasound-assisted extraction and quantitative analysis by HPLC-DAD (**A**); chemical structures of four ginsenosides quantified in this study (**B**).

However, according to the HPLC chromatogram, ginsenoside Rc cannot be resolved from adjacent peaks, which led to difficulties in quantification analysis. In addition, UPLC-Q-TOF-MS was used to analyze the 70% ethanol extract. The TIC chromatogram and the identification of chromatographic peaks are shown in Supplementary Materials Figure S4 and Supplementary Materials Table S2. Supplementary Materials Figure S5 shows the TIC chromatogram of mixed reference solution of five ginsenosides. Supplementary Materials Figure S6 shows the secondary mass spectrum of ginsenosides of the DES5 extract. According to the results and based on the TIC of mixed reference solution of five ginsenosides, the fragment ions obtained by primary mass spectrometry data and secondary mass spectrometry, combined with the existing literature [34,35,36] and the database established by our group, five ginsenosides were identified, and eleven ginsenosides were tentatively characterized. Figure 1A,B show the MS/MS spectra of representative types of ginsenoside PPT (Rg_1_) and PPD (Rb_1_) as examples. In Figure 1A, the fragment ions at *m*/*z* 637.4321 and *m*/*z* 475.3788 were generated by the gradual loss of glucose residues. In Figure 1B, the fragment ions at *m*/*z* 945.5507, *m*/*z* 783.4951, *m*/*z* 621.4445 and *m*/*z* 459.3829 were generated by the gradual loss of glucose residues. We then combined the results obtained from HPLC and UPLC-Q-TOF-MS. The results demonstrate that the contents of less polar ginsenosides other than these five ginsenosides are not high enough to enable quantification. Therefore, four ginsenosides, namely Rg_1_, Re, Rf, and Rb_1_, were selected for subsequent analysis. Their chemical structures are indicated in Figure 2B.

### 3.2. Synthesis of DESs

DESs are generated from HBAs and HBDs via hydrogen bonds. For the preparation of DESs, common methods include heating and stirring, freeze-drying, and vacuum evaporation techniques [11,15]. In the present research, the heating and stirring methods were utilized since they are simple approaches and can adopt different temperatures, varying from room temperature to approximately 130 °C, depending on the melting points and stability of the substance [10,15].

Based on previous reports [6,15,16], the present research combined inexpensive and readily available HBAs and HBDs in different ratios with good safety and biodegradability to prepare binary and ternary DESs. As a result, eighteen different DESs, including three acid-based DESs, two amine-based DESs, three glycosyl-based DESs, five alcohol-based DESs, and five ternary DESs, were prepared as clear and transparent liquids, which were labeled DES1 to DES18, respectively. In addition, DES(DES0), consisting of glycerol, L-proline, and sucrose in a ratio of 9:4:1, developed by Jeong KM et al. [16], was prepared and employed for comparison. All the resulting DESs prepared are indicated in Table 1.

### 3.3. Screening of DESs for Extraction of Ginsenosides

**Figure 3:**
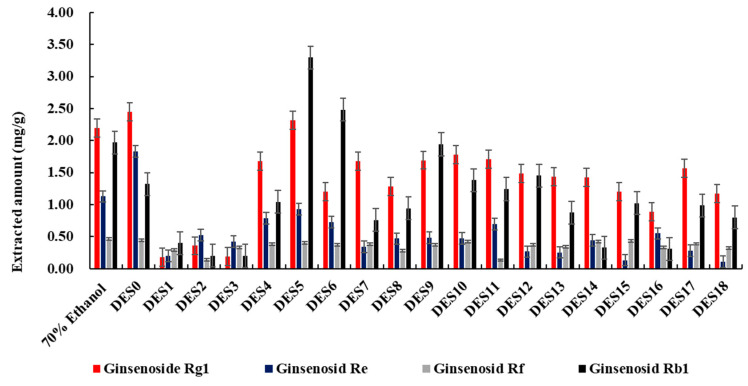
Extraction amounts of the 18 prepared deep eutectic solvents in comparison to 70% ethanol (*n* = 3).

Figure 2A shows the overall scheme for the extraction and analysis of four ginsenosides from the original plants in this research. Due to the viscosities of DESs, water is usually added to enable easier handling [15,16,17,18]. In this study, for the purpose of initial DES screening, all the prepared DESs were blended with water at a ratio of 7:3. A comparison of the extraction efficiencies of the DESs with 70% ethanol is shown in Figure 3.

**Figure 4:**
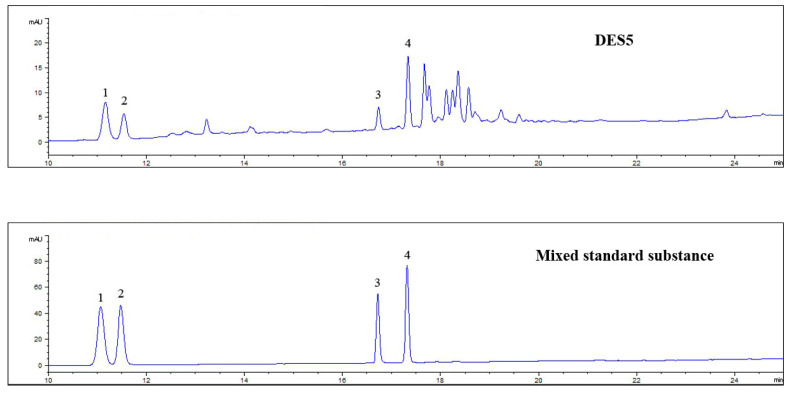
HPLC chromatograms of deep eutectic solvent-5 (1~4 corresponds to ginsenoside Rg_1_, Re, Rf, Rb_1_).

The results demonstrate that amine-based DESs perform better than other blends. Among all the screened solvents, DES5, which consists of choline chloride and urea at a ratio of 1:2, displays the highest extraction efficiency. Figure 4 illustrates the HPLC chromatogram for DES5. The extracted amount of DES5 is higher than that of 70% ethanol and DES0, especially for the Rg_1_ and Rb_1_ ginsenosides. In addition, the summed amounts for the four ginsenosides were compared. The extracted amount for DES5 is 20% higher than that for 70% ethanol. This may be due to a better hydrophilicity and antioxidant activity than that of the other solvents [38,39]. It is known that hydrogen bonding is one of the most important interactions to improve the solubility of compounds during extraction based on DESs [15,16]. When ginsenosides are dissolved in DESs, they form hydrogen bonds with DESs to enable extraction and enrichment. Polar ginsenosides are easily soluble in hydrophilic solvents, which promotes the formation of hydrogen bonds. Thus, the extraction efficiency is increased.

In addition, the results indicate that acid-based DESs yield remarkably lower extraction efficiency than other blends. The properties of HBDs are known to affect the acidity of DESs [13]. Therefore, the pH of the DESs was tested. As shown in Supplementary Materials Table S3, three acid-based DESs, ChCl: Lac, Pro: Lac, and Bet: Lac, exhibit moderate acidity, while the other solvents exhibit neutral values. Thus, the above results allow us to conclude that acid-based DESs are unsuitable for the extraction of neutral ginsenosides because of their acidity. Thus, the pH of the solvents has a great significance in the extraction of ginsenosides.

For the glycosyl-based DESs, alcohol-based DESs, and ternary DESs, their extraction amounts are all lower than that of 70% ethanol. Concerning glycosyl-based and alcohol-based DESs, the hydrogen bond between the hydroxyl group and HBAs is weaker than that formed between the amide group and HBAs. Thus, the polarity of the glycosyl-based DESs and alcohol-based DESs is less than that of the amine-based DESs, resulting in lower extraction efficiency. Ternary DESs consist of glycerol, HBAs, and glycosyl-based or alcohol-based HBDs. The purpose of the addition of glycerol is to reduce the viscosity of DESs [17]. The results reveal that the extraction efficiency of DESs is not significantly affected by glycerol. The principle underlying this phenomenon needs further study.

Therefore, DES5 was used as the final extraction medium for further analysis and optimization of the extraction conditions.

### 3.4. Qualitative Analysis of Extracted Ginsenosides

**Figure 5:**
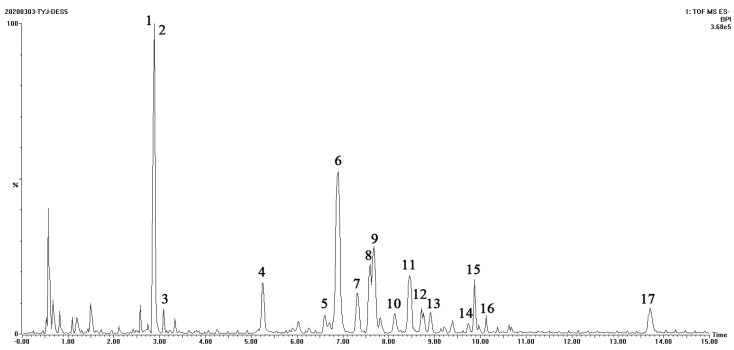
UHPLC−Q−TOF−MS TIC of ginsenosides recovered from deep eutectic solvent−5 extract (1–17 are ginsenoside Re, ginsenoside Rg_1_, acetyl-ginsenoside Rg_1_, ginsenoside Rf, ginsenoside Rg_2_, ginsenoside Rb_1_, M-ginsenoside Rb_1_, ginsenoside Ro, ginsenoside Rc, M-ginsenoside Rc, ginsenoside Rb_2_, ginsenoside Rb_3_, M-ginsenoside Rb_2_, M-ginsenoside Rb_3_, ginsenoside Rd, M-ginsenoside Rd and chikusetsusaponin IVa).

After the SPE of the DES5 extract, qualitative analysis of the extracted ginsenosides was performed. For the analysis of extracted ginsenosides, UPLC-Q-TOF-MS was employed. Figure 5 and Table 2 illustrate the TIC chromatogram and the identification of chromatographic peaks. Supplementary Materials Figure S5 shows the TIC chromatogram of mixed reference solution of five ginsenosides. Supplementary Materials Figure S6 shows the secondary mass spectrum of ginsenosides of the DES5 extract. According to the results and the fragment ions obtained by primary mass spectrometry data and secondary mass spectrometry, combined with the existing literature [34,35,36] and the database established by our group, five ginsenosides were identified, and eleven ginsenosides were tentatively characterized, which included six PPT, ten PPD, and one OA. Among them, one acetylated ginsenoside and five malonyl ginsenosides are included. The chromatographic peak attribution corresponds to that of conventional solvents. The qualitative analysis results prove that the green extraction method for ginsenosides is effective and feasible.

### 3.5. Investigation and Optimization of Extraction Conditions

The conditions that may affect the extraction efficiency were investigated and optimized to achieve the highest efficiency for ginsenoside extraction. According to the literature and our previous study [16,29], three variables, including the DES content in the extraction solvent (20~80%), liquid/solid ratios (5~25 mL g^−1^), and ultrasound extraction time (15~120 min), were evaluated for optimization.

**Figure 6:**
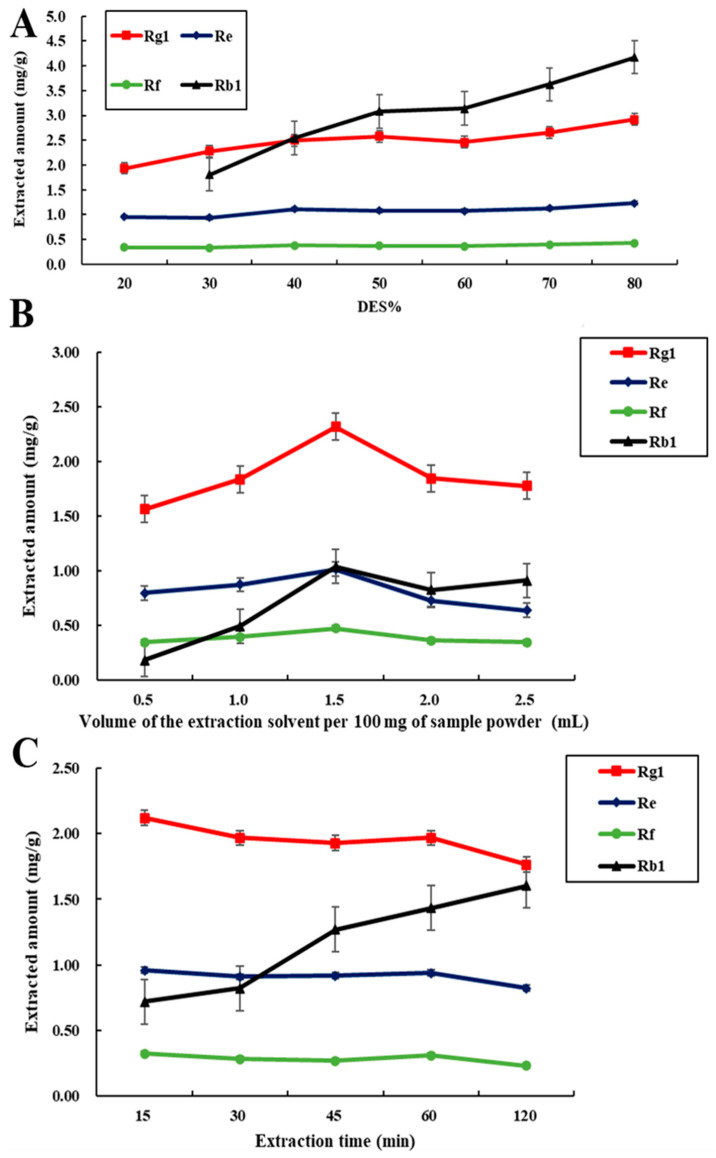
Effect of the deep eutectic solvent content in the extraction solvent (**A**); the liquid/solid ratio (**B**); and the extraction time (**C**) on extracted amounts of ginsenosides (*n* = 3).

Due to the viscosity of the DESs, water was added to the solvent for extraction [15,16]. The content of water normally affects the extraction efficiency. Therefore, we investigated the extraction effect for 20−80% DES5. As shown in Figure 6A, with increasing water content in DES5, the amount of ginsenosides extracted decreased. According to the Law of Similar Mutual Solubility, the greater the polarity of the solvent, the better the extraction effect of polar ginsenosides. Thus, as shown in Supplementary Materials Figure S3, higher-polarity aqueous methanol and ethanol are more efficient than pure methanol and ethanol for extraction. It has been reported that a high water content in DES aqueous solutions is appropriate for the extraction of polar compounds [13,14,15]. The experimental results indicate that 20 wt% DES5 aqueous solutions displays the best extraction performance. This may be attributed to hydrogen bonds. As the water content is increased, the interaction of HBAs and HBDs is affected, which leads to a decrease in the extraction amount. Therefore, excessive water content is not conducive to extraction.

Furthermore, the solid/liquid ratios and ultrasound time were investigated. The extraction efficiency of different liquid-to-solid ratios was investigated by adding various volumes of solvent to 100 mg of white ginseng powder. As shown in Figure 6B, the extraction yields are increased when the solid ratios are in the range of 5 mL g^−1^ to 15 mL g^−1^ and then decreased when the solid ratios range from 15 mL g^−1^ to 25 mL g^−1^. Generally, with increasing amount of solvent, the contact between the target components and solvent is also strengthened, which increases the leaching rate. However, an excess amount of solvent enhances the hydrogen bonds formed between HBAs and HBDs, which weakens the hydrogen bonds formed between the solvent and target molecule, resulting in a lower extraction efficiency. Thus, a solid/liquid ratio of 15 mL g^−1^ was considered to be the optimum. For the extraction time, a range of 15 min to 120 min was evaluated. As shown in Figure 6C, the extracted amounts decrease as the extraction time is increased. With a continuous increase in the ultrasonic extraction time, the already formed hydrogen bonds c be disrupted, resulting in reduced extraction efficiency. Thus, an ultrasound time of 15 min demonstrated the best performance and was selected as the optimal condition.

Therefore, the optimal conditions were 80% DES5, 15 mL g^−1^ liquid to solid ratio, and ultrasonic extraction for 15 min. The extraction efficiency of the optimized DES5-based method was evaluated, and the extracted amounts of ginsenoside Rg_1_, Re, Rf, and Rb_1_ were 2.70 mg g^−1^, 1.03 mg g^−1^, 1.57 mg g^−1^, and 2.25 mg g^−1^, respectively. After optimization, based on the total amount of four ginsenosides, the extracted amount of DES5 was 31% higher than that of 70% ethanol. Thus, our green extraction method for ginsenosides is proven to be efficient.

### 3.6. Method Validation

Under optimized conditions, the established method was evaluated using the working curve and other analytical properties, such as linearity, sensitivity, precision, and recovery. The working curve was drawn by plotting the relationship between the peak area value and the concentration of the ginsenoside standards in the mixed reference solution. The results are shown in Table 3 and Table 4. Good working curve linearity and correlation coefficients (r) were obtained. The RSDs for the precision ranged from 0.87% to 3.78%. The recovery of the ginsenosides ranged from 95.0% to 108.2%. In addition, our detection method has good specificity for ginsenosides on the basis of previous study. The experimental results showed that the optimized DES-UAE method achieves good accuracy and precision for the determination of ginsenosides.

### 3.7. Comparison of the DES-UAE Method with Other Methods

For further evaluation, we compared the green extraction method established in this study with existing commonly used extraction methods. As shown in Table 5, our method exhibits the best extraction efficiency with the shortest pretreatment and detection times. In addition, our method successfully replaces organic solvents with deep eutectic solvents, which achieves green and efficient extraction of ginsenosides.

## 4. Conclusions

An ultrasonic-assisted extraction method with deep eutectic solvents coupled with HPLC-DAD was established for the analysis of ginsenosides from *P. ginseng*. Among all the prepared solvents, a binary DES combined with choline chloride and urea at a ratio of 1:2 display the best extraction efficiency. The entire extraction process from solvent preparation to HPLC-UV analysis only takes approximately 3 h, which greatly reduces the extraction time. SPE was employed to recover ginsenosides from DES5 extract and is proven to be feasible. This research supplies instruction for further research into DES-based green extraction methods. Theoretically, this will enable us to develop new methods for extracting active components from natural products based on green solvents.

## Figures and Tables

**Table 1:** Components of deep eutectic solvents used in the experiments.

Number	Deep Eutectic Solvents	Mole Ratio
1	Choline chloride: lactic acid	1:4
2	L-proline: lactic acid	1:4
3	Betaine: lactic acid	1:4
4	Betaine: urea	1:2
5	Choline chloride: urea	1:2
6	L-proline: D-(+)-maltose	2:1
7	Betaine: D-(+)-maltose	1:1
8	Choline chloride: D-(+)-maltose	2:1
9	Betaine: ethylene glycol	1:4
10	Betaine:1,4-butanediol	1:4
11	Choline chloride: 1,2-propanediol	1:4
12	Betaine:1,2-propanediol	1:4
13	Choline chloride: D-sorbitol	1:1
14	Glycerol: Choline chloride: D-sorbitol	1:0.5:0.5
15	Glycerol: Choline chloride: D-(+)-maltose	5:4:1
16	Glycerol: L-proline: D-(+)-maltose	5:4:1
17	Glycerol: Betaine: D-(+)-maltose	5:1:1
18	Glycerol: Choline chloride: D-(+)-glucose	5:1:1

**Table 2:** Compounds identified from ginsenosides recovered from deep eutectic solvent-5 extract.

Number	Retention Time	Identity	Molecular Formula	Molecule Weight	[M−H]^−^ Measured Value	Mass Fragment
				*m*/*z*	*m*/*z*	
1	2.87	Ginsenoside Re	C_48_H_82_O_18_	946.5501	945.5577	783.4982, 637.4429, 475.3860
2	2.87	Ginsenoside Rg_1_	C_42_H_72_O_14_	800.4922	799.4974	637.4429, 475.3853
3	3.08	Acetyl-Ginsenoside Rg_1_	C_44_H_74_O_15_	842.5028	841.5055	637.4424, 475.3689, 179.0534, 161.0432
4	5.24	Ginsenoside Rf	C_42_H_72_O_14_	800.4922	799.4948	637.4409, 475.3872, 391.3035
5	6.59	Ginsenoside Rg_2_	C_42_H_72_O_13_	784.4973	783.4976	637.4370, 475.3929, 391.2038
6	6.88	Ginsenoside Rb_1_	C_54_H_92_O_23_	1108.6029	1107.6194	945.5510, 783.4947, 621.4453, 459.3857
7	7.31	M-Ginsenoside Rb_1_	C_57_H_94_O_26_	1194.6033	1193.6294	1107.6102, 945.5532, 783.4930, 621.4282, 459.3890
8	7.65	Ginsenoside Ro	C_48_H_76_O_19_	956.4981	955.5157	793.4459, 455.3476
9	7.65	Ginsenoside Rc	C_53_H_90_O_22_	1078.5924	1077.6031	945.5517, 783.4988, 621.4410, 459.3914
10	8.12	M-Ginsenoside Rc	C_56_H_92_O_25_	1164.5928	1163.6096	1077.5968, 945.5502, 783.4940, 621.4370, 459.4090
11	8.46	Ginsenoside Rb_2_	C_53_H_90_O_22_	1078.5924	1077.6072	945.5524, 783.4978, 621.4457
12	8.71	Ginsenoside Rb_3_	C_53_H_90_O_22_	1078.5924	1077.5988	945.5562, 783.5023, 621.4474
13	8.90	M-Ginsenoside Rb_2_	C_56_H_92_O_25_	1164.5928	1163.6073	1077.5972, 945.5626, 783.5019, 621.4164, 459.3085
14	9.75	M-Ginsenoside Rb_3_	C_56_H_92_O_25_	1164.5928	1163.6047	1077.5912, 945.5482, 783.4807, 621.4417
15	9.87	Ginsenoside Rd	C_48_H_82_O_18_	946.5501	945.5523	783.4973, 621.4481, 459.4015
16	10.12	M-Ginsenoside Rd	C_51_H_84_O_21_	1032.5505	1031.5533	945.5515, 783.5026, 621.4420, 459.3722
17	13.70	Chikusetsusaponin Iva	C_42_H_66_O_14_	794.4453	793.4451	631.3763, 455.3561

**Table 3:** The regression equations and the precision of the method.

Analytes	Regression Equation	Linear Range (μg mL^−1^)	Correlation Coefficient (r)	LOD (μg mL^−1^)	LOQ (μg mL^−1^)	Precision (%)
Ginsenoside Rg_1_	y = 1.3862x + 1.3295	25.3–404.0	0.9999	3.897	12.990	0.87
Ginsenoside Re	y = 1.1635x + 1.9422	25.6–410.0	1.0000	3.345	11.150	3.78
Ginsenoside Rf	y = 2.8179x + 4.6915	6.6–105.0	0.9999	1.602	5.339	0.96
Ginsenoside Rb_1_	y = 1.0523x + 0.2323	25.3–404.0	1.0000	1.524	5.081	2.70

**Table 4:** The recovery of the analytes.

Analytes	Initial Content (μg)	Added Content (μg)	Found (μg)	Recovery (%)
Ginsenoside Rg_1_	251.3	152.6	416.4	108.2
Ginsenoside Re	181.1	97.8	278.4	99.5
Ginsenoside Rf	62.2	40.0	100.9	96.8
Ginsenoside Rb_1_	237.4	120.7	352.1	95.0

**Table 5:** Comparison of the optimized method with other methods.

Method	Pretreatment Time (h)	Detection Time (min)	Extraction Amount (mg g^−1^)
Soxhlet extraction [37]	24	100	7.51
Ultrasonic extraction with 70% Ethanol	2	40	8.49
Ultrasonic extraction with deep eutectic solvent-0 [17]	24	55	6.10
Our optimized method	2	40	11.41

## Data Availability

Data are contained within the article or Supplementary Materials.

## Supplementary Materials

The following supporting information can be downloaded at: https://www.mdpi.com/article/10.3390/molecules27144339/s1, Figure S1: Chemical structures of four types of ginsenosides; Figure S2: HPLC chromatograms of the reference solvents; Figure S3: The extraction efficiency of the reference solvents; Figure S4: UHPLC–Q–TOF–MS TIC of the 70% ethanol extract; Figure S5: UHPLC–Q–-TOF–MS TIC of mixed reference solution of five ginsenosides; Figure S6: Secondary mass spectrum of ginsenosides of the DES5 extract; Table S1: Peak areas of four ginsenosides extracted by five conventional solvents; Table S2: Compounds identified from 70% ethanol extract; Table S3: pH values of the prepared deep eutectic solvents.
