# Supplementary material for: Development of Green and Efficient Extraction of Bioactive Ginsenosides from Panax ginseng with Deep Eutectic Solvents
Source: Molecules. 2022 Jul 6;27(14):4339. doi: 10.3390/molecules27144339 (PMC9315546; doi:10.3390/molecules27144339)
Supplement: Supplementary file 1 [file molecules-27-04339-s001.zip › molecules-1774081-Supplementary.pdf]

Supplementary Information

Development of Green and Efficient Extraction of Bioactive Ginsenosides from *Panax ginseng* with Deep Eutectic Solvents

Yujia Tu [1, 2], Linnan Li [1, *], Wenxiang Fan [1], Longchan Liu [1], Zhengtao Wang [1], Li Yang [1, 2, *]

1. The MOE Key Laboratory of Standardization of Chinese Medicines, the SATCM Key Laboratory of New Resources and Quality Evaluation of Chinese Medicines, Shanghai Key Laboratory of Compound Chinese Medicine, Institute of Chinese Materia Medica, Shanghai University of Traditional Chinese Medicine, Shanghai 201203, China

2. Shanghai Frontiers Science Center of TCM Chemical Biology, Institute of Interdisciplinary Integrative Medicine Research, Shanghai University of Traditional Chinese Medicines, Shanghai, 201203, China

*Correspondence

Prof. Li Yang, E-mail: yl7@shutcm.edu.cn; Tel: +86-021-51322506.

Prof. Linnan Li, E-mail: linnanli@shutcm.edu.cn; Tel: +86-021-51322506.

Supplementary Table S1. Peak areas of four ginsenosides extracted by five conventional solvents.

Extracting solvents / Peak area (mAU*s)	Water	100% Methanol	70% Methanol	100% Ethanol	70% Ethanol
Ginsenoside Rg_1	270.8	262.1	295.6	86.8	305.0
Ginsenoside Re	93.5	104.6	122.2	24.0	140.7
Ginsenoside Rf	31.1	74.4	84.4	25.8	86.1
Ginsenoside Rb_1	185.4	162.0	217.7	28.7	190.8

Supplementary Table S2. Compounds identified from 70% ethanol extract.

ID	Retention time	Identity	Molecular formula	Molecule weight m/z	$[M-H]^-$ measured value m/z	Mass fragment
1	2.96	Ginsenoside Re	$C_{48}H_{82}O_{18}$	946.5501	945.5529	783.4919, 637.4390, 475.3834
2	2.96	Ginsenoside Rg_1	$C_{42}H_{72}O_{14}$	800.4922	799.4937	637.4390, 475.3841, 391.2740
3	3.19	Acetyl-Ginsenoside Rg_1	$C_{44}H_{74}O_{15}$	842.5028	841.5056	637.4444, 475.3881, 179.0485, 161.0403
4	5.47	Ginsenoside Rf	$C_{42}H_{72}O_{14}$	800.4922	799.4935	637.4383, 475.3896, 391.2953
5	6.90	Ginsenoside Rg_2	$C_{42}H_{72}O_{13}$	784.4973	783.4999	637.4384, 475.3897, 391.2898
6	7.19	Ginsenoside Rb_1	$C_{54}H_{92}O_{23}$	1108.6029	1107.7627	945.5528, 783.4989, 621,4333, 459.3954
7	7.63	M-Ginsenoside Rb_1	$C_{57}H_{94}O_{26}$	1194.6033	1193.6381	1149.6182, 1107.6115, 945.5476, 783.4973, 621.4464
8	7.89	Ginsenoside Ro	$C_{48}H_{76}O_{19}$	956.4981	955.4877	793.4426, 455.3723
9	7.98	Ginsenoside Rc	$C_{53}H_{90}O_{22}$	1078.5924	1077.6038	945.5514, 783.4944, 621.4468, 459.3778
10	8.46	M-Ginsenoside Rc	$C_{56}H_{92}O_{25}$	1164.5928	1163.6204	1077.6021, 945.5532, 783.5054, 621.4446, 459.3960
11	8.78	Ginsenoside Rb_2	$C_{53}H_{90}O_{22}$	1078.5924	1077.7112	945.5515, 783.4916, 621.4460
12	9.04	Ginsenoside Rb_3	$C_{53}H_{90}O_{22}$	1078.5924	1077.6298	945.5428, 783.4963, 621.4475
13	9.18	M-Ginsenoside Rb_2	$C_{56}H_{92}O_{25}$	1164.5928	1163.6027	1077.5986, 945.5522, 783.4985, 621.4526, 459.3629
14	9.91	M-Ginsenoside Rb_3	$C_{56}H_{92}O_{25}$	1164.5928	1163.6047	1077.5974, 945.5388, 783.5089, 621.4965
15	10.04	Ginsenoside Rd	$C_{48}H_{82}O_{18}$	946.5501	945.5544	783.4977, 621.4473, 459.3713
16	10.26	M-Ginsenoside Rd	$C_{51}H_{84}O_{21}$	1032.5505	1031.5574	945.5580, 783.4990, 621.4485, 459.3925

Supplementary Table S3. pH values of the prepared deep eutectic solvents.

Number	Chemical composition	pH values
1	Choline chloride: lactic acid 1:4	4
2	L-proline: lactic acid 1:4	4
3	Betaine: lactic acid 1:4	4
4	Betaine: urea 1:2	7
5	Choline chloride: urea 1:2	7
6	L-proline: D-(+)-maltose 2:1	7
7	Betaine: D-(+)-maltose 1:1	7
8	Choline chloride: D-(+)-maltose 2:1	7
9	Betaine: ethylene glycol 1:4	7
10	Betaine:1,4-butanediol 1:4	7
11	Choline chloride: 1,2-propanediol 1:4	7
12	Betaine:1,2-propanediol 1:4	7
13	Choline chloride: D-sorbitol 1:1	7
14	Glycerol: Choline chloride: D-sorbitol 1:0.5:0.5	7
15	Glycerol: Choline chloride: D-(+)-maltose 5:4:1	7
16	Glycerol: L-proline: D-(+)-maltose 5:4:1	7
17	Glycerol: Betaine: D-(+)-maltose 5:4:1	7
18	Glycerol: Choline chloride: D-(+)-glucose 5:4:1	7

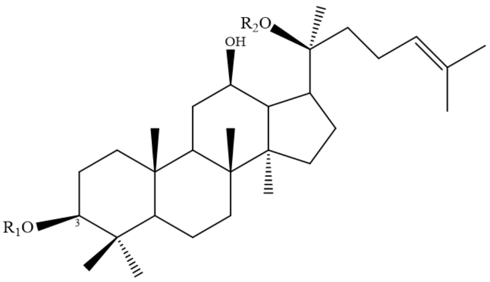

PPD Type

**Ginsenoside Rb$_1$**	**R$_1$=-Glc(2→1)-Glc**	**R$_2$=-Glc(6→1)-Glc**
**Ginsenoside Rb$_2$**	**R$_1$=-Glc(2→1)-Glc**	**R$_2$=-Glc(6→1)-Ara(*p*)**
**Ginsenoside Rb$_3$**	**R$_1$=-Glc(2→1)-Glc**	**R$_2$=-Glc(6→1)-Xyl**
**Ginsenoside Rc**	**R$_1$=-Glc(2→1)-Glc**	**R$_2$=-Glc(6→1)- Ara(*f*)**
**Ginsenoside Rd**	**R$_1$=-Glc(2→1)-Glc**	**R$_2$=-Glc**
**m-Ginsenoside Rb$_1$**	**R$_1$=-Glc(2→1)-Glc-6-malonyl**	**R$_2$=-Glc(6→1)-Glc**
**m-Ginsenoside Rb$_2$**	**R$_1$=-Glc(2→1)-Glc-6-malonyl**	**R$_2$=-Glc(6→1)- Ara(*p*)**
**m-Ginsenoside Rc**	**R$_1$=-Glc(2→1)-Glc-6-malonyl**	**R$_2$=-Glc(6→1)- Ara(*f*)**

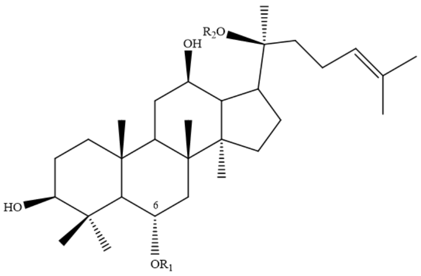

PPT Type

**Ginsenoside Rg$_1$**	**R$_1$=-Glc**	**R$_2$=-Glc**
**Ginsenoside Re**	**R$_1$=-Glc(2→1)-Rha**	**R$_2$=-Glc**
**Ginsenoside Rf**	**R$_1$=-Glc(2→1)-Glc**	**R$_2$=-H**

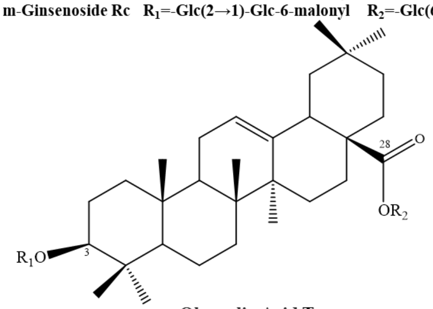

Oleanolic Acid Type

**Ginsenoside Ro**	**R$_1$=-Glc-UAO(2→1)-Glc**	**R$_2$=-Glc**
**Chikusetsusaponin Iva**	**R$_1$=-Glc-UAO**	**R$_2$=-Glc**

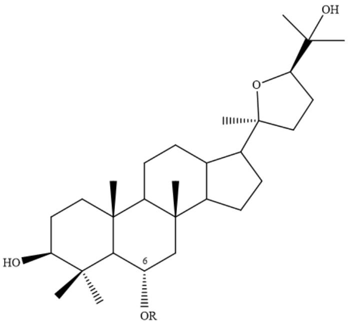

Ocotillol type

Pseudoginsenoside F$_{11}$ **R=-Glc-(2→1)-Rha**

Supplementary Figure. S1. Chemical structures of four types of ginsenosides.

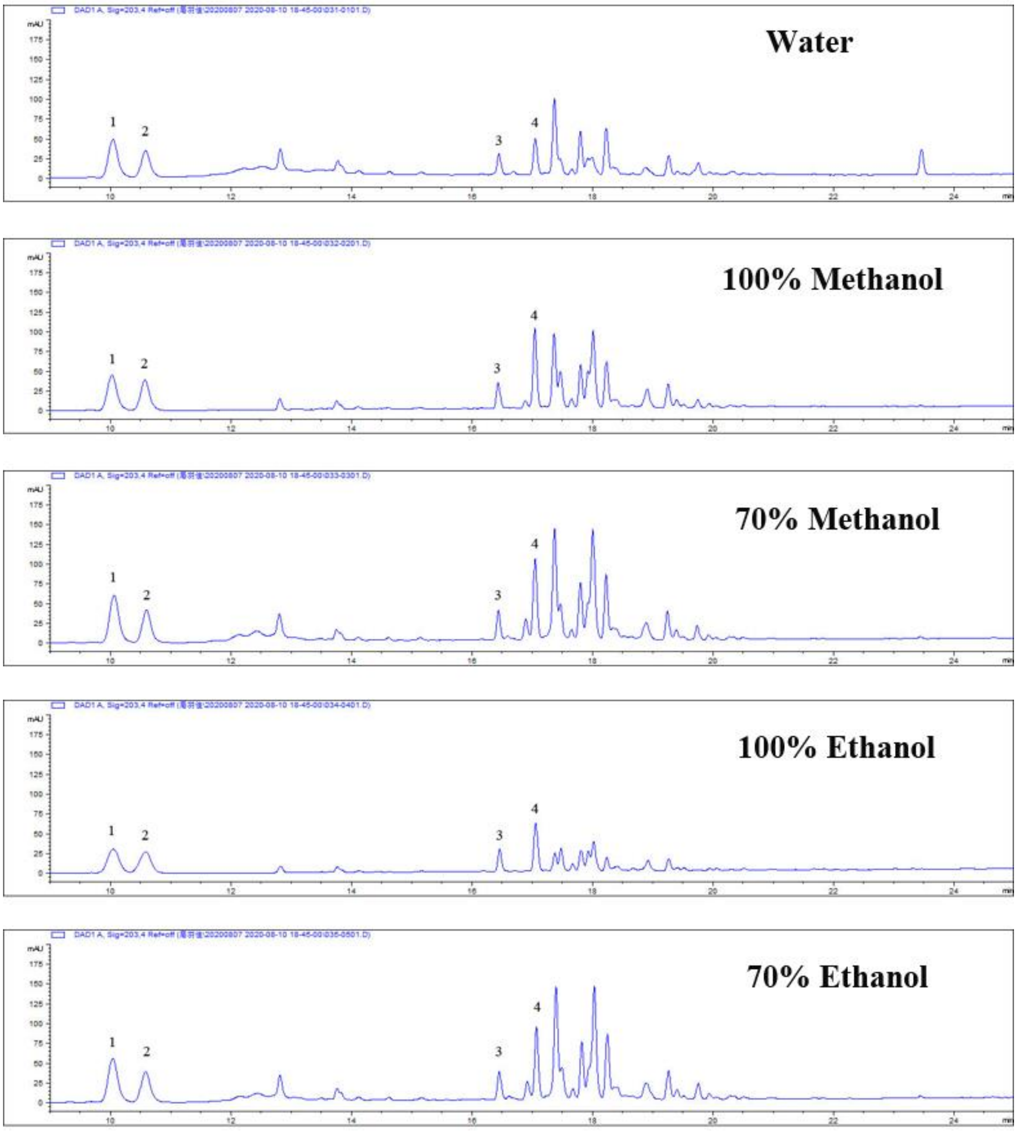

Supplementary Figure. S2. HPLC chromatograms of the reference solvents (1, ginsenoside Rg_1;2, ginsenoside Re;3, ginsenoside Rf; 4, ginsenoside Rb_1).

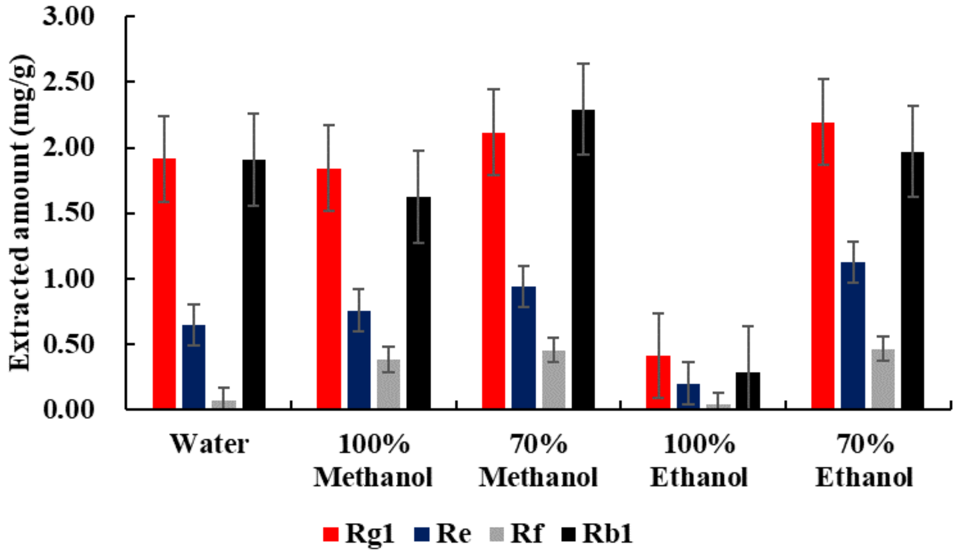

Supplementary Figure. S3. The extraction efficiency of the reference solvents.

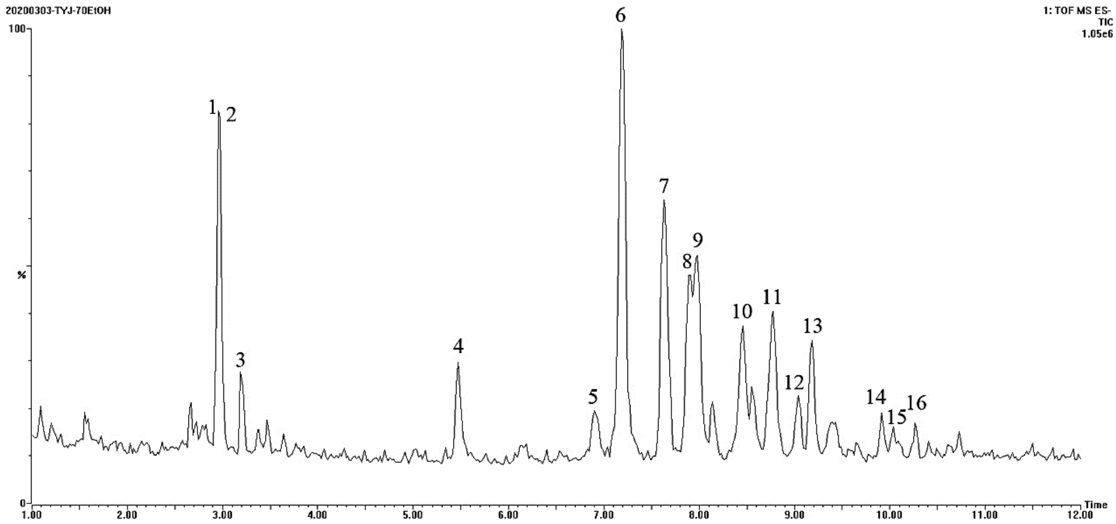

Supplementary Figure. S4. UHPLC–Q-TOF-MS TIC of the 70% ethanol extract (1-16 are ginsenoside Re, ginsenoside Rg_1, Acetyl-ginsenoside Rg_1, ginsenoside Rf, ginsenoside Rg_2, ginsenoside Rb_1, M-ginsenoside Rb_1, ginsenoside Ro, ginsenoside Rc, M-ginsenoside Rc, ginsenoside Rb_2, ginsenoside Rb_3, M-ginsenoside Rb_2, M-ginsenoside Rb_3, ginsenoside Rd and M-ginsenoside Rd).

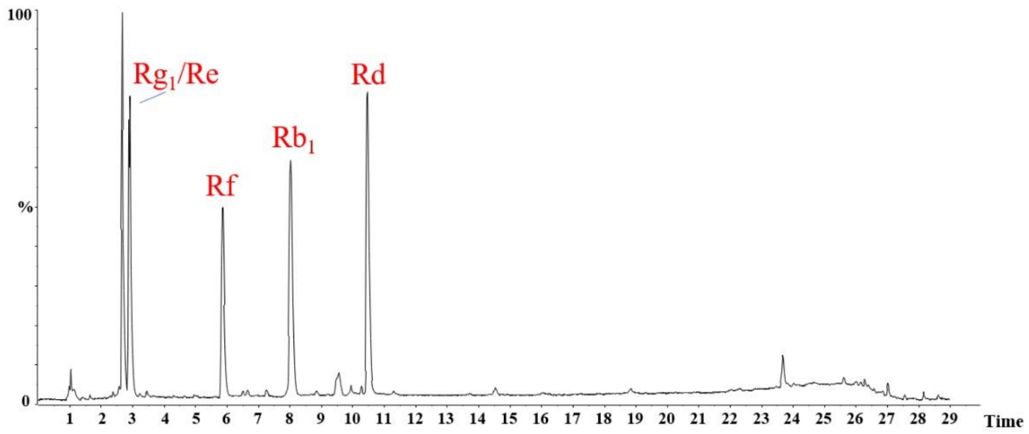

Supplementary Figure. S5. UHPLC–Q-TOF-MS TIC of mixed reference solution of five ginsenosides.

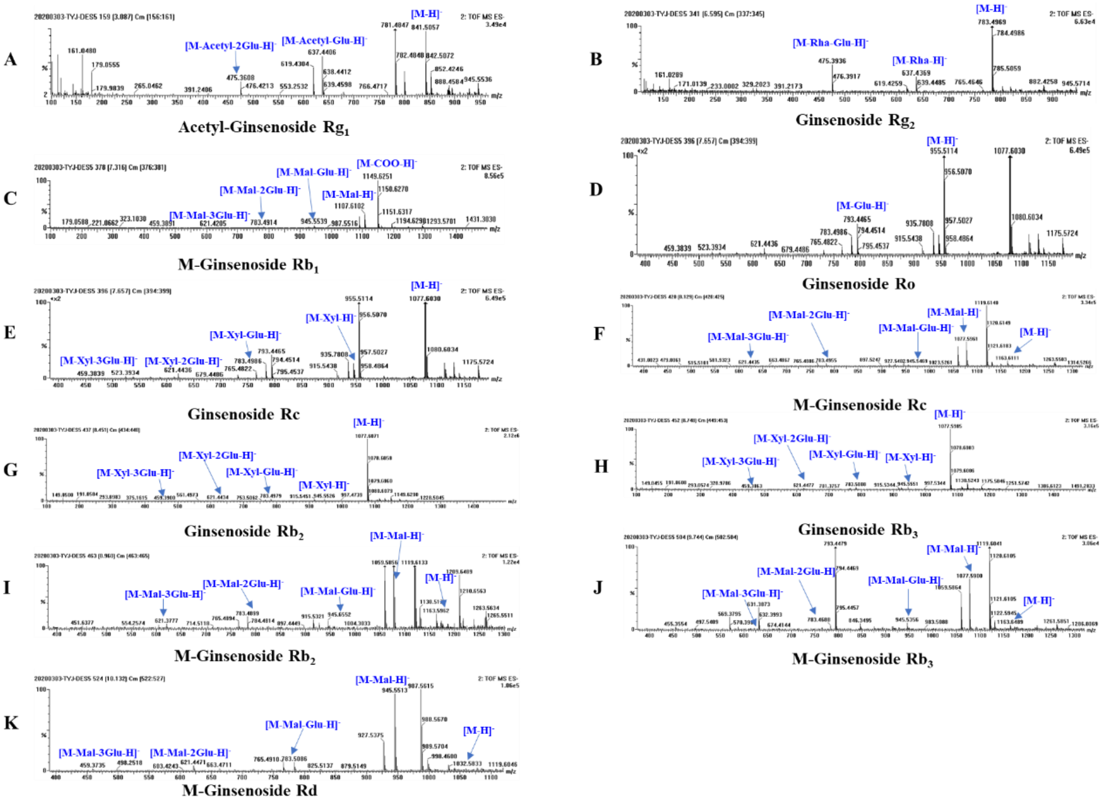

Supplementary Figure. S6. Secondary mass spectrum of ginsenosides of the DES5 extract (A-K are Acetyl-ginsenoside Rg_1, ginsenoside Rg_2, M-ginsenoside Rb_1, ginsenoside Ro, ginsenoside Rc, M-ginsenoside Rc, ginsenoside Rb_2, ginsenoside Rb_3, M-ginsenoside Rb_2, M-ginsenoside Rb_3 and M-ginsenoside Rd).
